# Effectiveness of olanzapine in the treatment of anorexia nervosa: A systematic review and meta‐analysis

**DOI:** 10.1002/brb3.2498

**Published:** 2022-01-12

**Authors:** Ruijun Han, Qingtao Bian, Hao Chen

**Affiliations:** ^1^ Department of Psychiatry Beijing Huilongguan Hospital Beijing China; ^2^ Department of Internal Medicine Teikyo University Hospital Tokyo Japan

**Keywords:** adjuvant treatment, anorexia nervosa, body mass index, olanzapine, pharmacotherapy

## Abstract

**Objective:**

Anorexia nervosa (AN) is a severe psychiatric disorder characterized by starvation and malnutrition, a high incidence of coexisting psychiatric conditions, and treatment resistance. The effect of pharmacotherapy has been controversial.

**Method:**

A systematic review was conducted for evidence of an effect of olanzapine versus placebo in adults or its effect as adjuvant treatment of AN in adolescents.

**Results:**

A total of seven articles (304 patients with AN) were identified. There were four double‐blind, randomized studies examining the effect of olanzapine in the treatment of AN. The mean difference in body mass index (BMI) at the end of treatment between olanzapine and placebo was 0.67 kg/m^2^ (95% confidence interval (CI) 0.15–1.18 kg/m^2^; *p* = 0.01; *I* ^2^ = 0%, *p* for heterogeneity < 0.79). The olanzapine groups showed a significant increase in BMI of 0.68 kg/m^2^ (95% CI 0.22–1.13 kg/m^2^; *p* < 0.001; *I* ^2^ = 0%, *p* for heterogeneity = 0.74) compared to the placebo groups. Only two studies examined the effect of olanzapine as adjuvant treatment in adolescents and showed an increase in BMI of 0.66 kg/m^2^ (95% CI −0.36 to 1.67 kg/m^2^; *p* = 0.21; *I* ^2^ = 11%, *p* for heterogeneity = 0.32).

**Discussion:**

Olanzapine showed efficacy in the treatment of AN with an increased BMI at the end of treatment in adults. The effect of olanzapine as adjuvant treatment in adolescents remains unclear.

## Abstract 2

Olanzapine was effective in the treatment of adult anorexia nervosa. Compared with placebo, olanzapine treatment resulted in an increase in BMI, 0.6 kg/m^2^, at the end of treatment. The effect of olanzapine as adjuvant treatment in adolescents remains unclear.

## 1 INTRODUCTION

Anorexia nervosa (AN) is a severe psychiatric disorder characterized by starvation and malnutrition, a high incidence of coexisting psychiatric conditions, treatment resistance, and a substantial risk of death from medical complications and suicide (Mitchell & Peterson, 2020). Patients have an intense fear of gaining weight and a distorted body image, with the inability to recognize the seriousness of their significantly low body weight (Moore & Bokor, 2021). The aetiology of AN is unknown, although it has been described as multifactorial. The treatment of AN is challenging, the high rates of relapse following successful efforts at weight restoration. There are several national guidelines for the treatment of AN published in Canada, the USA, UK (NICE guidelines), and Denmark. They all aim at treating the severe underweight, restoring nutritional intake, and treating psychological symptoms and behavioural signs associated with the disorder (Rosager et al., 2021).

There is an extensive history of medication trials for the pharmacological treatment of AN, based on the initial observation that individuals with AN exhibit core symptoms that are believed to suggest the presence of a biological disturbance. Given the near delusional quality in AN of some of the beliefs around food, weight, and body image, together with rigidity, obsessionality, and intense anxiety characteristic of AN, antipsychotic medications have also been proposed as potential therapeutic agents for AN. Several randomized, double‐blind, placebo‐controlled trials demonstrated both a greater rate of weight gain and higher BMI at the end of treatment (Attia et al., 2011, 2019; Bissada et al., 2008).

For children and adolescents with AN, the major guidelines recommend family‐based treatment. The treatment of choice for young adults and adults with AN is the Maudsley Anorexia Nervosa Treatment for Adults (MANTRA), Cognitive Behavior Therapy‐Enhanced (CBT‐E), and Specialist Supportive Clinical Management (SSCM), but none of these treatments seems to be superior (Jansingh et al., 2020). Adjunctive olanzapine treatment for AN in adolescents was also reported recently (Norris et al., 2011; Spettigue et al., 2018).

A meta‐analysis and systematic review of olanzapine were conducted recently (Meftah et al., 2020; Murray et al., 2019). Due to the limited number of studies, the conclusion regarding olanzapine in the treatment of AN was still unclear. This systematic review attempted to identify all controlled clinical trials of olanzapine in AN patients and assessed the effect on weight gain. Adult and adolescent AN patients were analyzed separately.

## 2 METHODS

### 2.1 Overview

The study protocol followed the Meta‐analysis Of Observational Studies in Epidemiology (MOOSE) group statement and was registered in the University Hospital Medical Information Network (ID: UMIN000043186). (“Cooperative organization for national medical schools in Japan. University hospital Medical Information Network (UMIN) Center. Available at: https://www.umin.ac.jp/ctr/; Stroup et al., 2000) The need for institutional review board approval was waived since no human subjects or private information is being accessed.

### 2.2 Search strategy and selection criteria

Three major databases (Medline, CHAHL, and Web of Science) were searched on June 1, 2021. The two reviewers independently extracted and recorded data for a predefined checklist including the following items: study characteristics (i.e., country and year of study), characteristics of the cohort, and outcomes. The following search formula was used: ((anorexia nervosa) OR (eating disorder)) and (olanzapine). Two review authors (RH and HC) independently screened the titles and abstracts and carefully evaluated the full text to select eligible articles. In cases of discrepancy, they reached a consensus through discussion. Review articles and included original articles were hand‐searched (RH and HC) for additional research papers that met the inclusion criteria.

No restrictions were placed on article types or publication language. To be included, a study had to include: (1) patients with AN; (2) olanzapine treatment; and (3) weight gain after treatment could be calculated. Exclusion criteria were: (1) case report; and (2) single‐arm study.

### 2.3 Outcomes

The primary outcome was gain in weight. In placebo‐controlled studies, the difference in BMI at the end of treatment between olanzapine and placebo was examined. Increased BMI after treatment by olanzapine or placebo was also identified. In adolescents using olanzapine as adjuvant treatment, only increased BMI was analyzed, because only increased BMI was available in one study.

### 2.4 Quality assessment

Two reviewers independently assessed the methodological quality of selected studies using the Newcastle–Ottawa Scale quality assessment to evaluate the quality of observational studies (Stang, 2010). Disagreements among reviewers were discussed, with agreement reached by consensus.

### 2.5 Statistics

All analyses were performed using Review Manager version 5.3 (Cochrane Collaboration, Oxford, UK). Figures prepared using Review Manager were adjusted as necessary. Mean differences and 95% confidence intervals (95%CIs) of BMI were calculated before and after treatment with olanzapine or placebo separately. Then, increased BMI was compared between groups. Heterogeneity evaluated using *I* ^2^ statistics was interpreted as follows: *I* ^2^  =  0%, no heterogeneity; *I* ^2^ > 0% but < 25%, minimal heterogeneity; *I* ^2^ ≥ 25% but < 50%, mild heterogeneity; *I* ^2^ ≥ 50% but < 75%, moderate heterogeneity; and *I* ^2^ ≥ 75%, strong heterogeneity (Higgins et al., 2003).

### 2.6 Role of the funding source

There was no funding source for this study.

## 3 RESULTS

**FIGURE 1:**
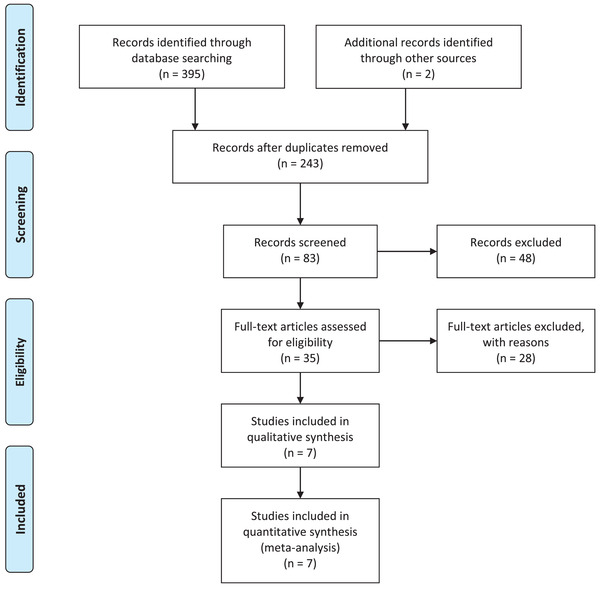
PRISMA flow chart for study selection

A total of 397 articles, including 395 articles through database searching and two articles by hand searching, were identified. There were 243, 83, and 7 articles left after removing duplication, screening, and full‐article reading, respectively (Figure 1). A total of 304 patients with AN were identified in this study (Table 1) (Attia et al., 2011, 2019; Bissada et al., 2008; Brambilla et al., 2007; Kafantaris et al., 2011; Norris et al., 2011; Spettigue et al., 2018). All studies were written in English. Five studies were conducted in the United States; the remaining studies were conducted in Italy and Canada each, including one multicenter study. Four studies examined the effect of olanzapine versus placebo in double‐blind, randomized studies in the treatment of AN. Three studies identified the effect of olanzapine as adjuvant treatment in adolescents. Newcastle–Ottawa Scale scores ranged from 6 to 7.

**TABLE 1:** Characteristics and backgrounds of included studies

Author	Year	Country	Research	Cases	Age, year(Standard Deviation)	Follow‐up	Subcategories	Primary Outcome	NOS
Attia	2011	USA	DB	23	27.7 (9.1)	8 weeks	AN or ANBP	BMI	7
Attia	2019	USA	DB	152	29 (10.9)	16 weeks	ANR or ANBP	BMI	7
Bissada	2008	Canada	DB	34	26.8 (9.2)	10 weeks	ANR or ANBP	BMI	7
Brambllia	2007	Italy	DB	30	25 (6.7)	12 weeks	ANR 18; ANBP 12	BMI	7
Kafantaris	2011	USA	DB	20	17.1^†^	10 weeks	ANR 20	BMI	7
Norris	2011	USA	MP	22	14.3 (1.8)	252 days	Unknown	BMI	6
Spettigue	2018	USA	OL	23	15.6 (1.5)	12 weeks	ANR 20; EDNOS 3	BMI	6

^†^ : range from 12·3−21·8 years; AN, anorexia nervosa subtype, ANBP, anorexia nervosa binge‐purge subtype; BMI, body mass index; DB, double‐blind; EDNOS‐R, eating disorder not otherwise specified restricting subtype; MP, matched pairs; NOS, Newcastle–Ottawa Scale; OL, open label.

**FIGURE 2:**
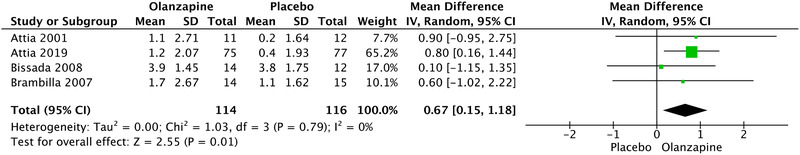
The difference in BMI at the end of treatment: olanzapine versus placebo

**FIGURE 3:**
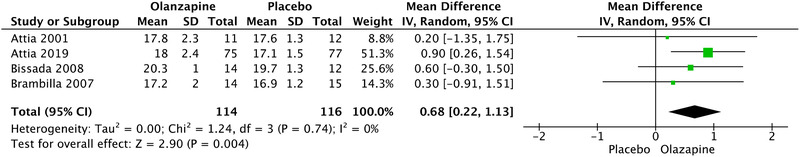
Increased BMI after treatment with olanzapine and placebo

After treatment with olanzapine, the mean difference in BMI at the end of treatment between olanzapine and placebo was 0.67 kg/m^2^ (95% confidence interval (CI) 0.15–1.18 kg/m^2^; *p* = 0.01; *I* ^2^ = 0%, *p* for heterogeneity = 0.79) (Figure 2). Although all of the above studies were double‐blind, randomized studies, due to the small sample sizes of several studies, the initial BMI might have been different before treatment. The increase in BMI after treatment was also compared between olanzapine and placebo. The effect of olanzapine and placebo in increasing BMI were calculated separately with 95%CI (Figures S1 and S2). The BMI increased 2.02 kg/m^2^ (95%CI 0.48–3.55 kg/m^2^) after the treatment of olanzapine. An estimate increase of BMI was 0.19 kg/m^2^ (95%CI 0.12–0.25 kg/m^2^) weekly. Compared with placebo, the olanzapine group showed a significant increase in BMI of 0.68 kg/m^2^ (95%CI 0.22–1.13 kg/m^2^; *p* = 0.004; *I* ^2^ = 0%, *p* for heterogeneity = 0.74) (Figure 3).

**FIGURE 4:**
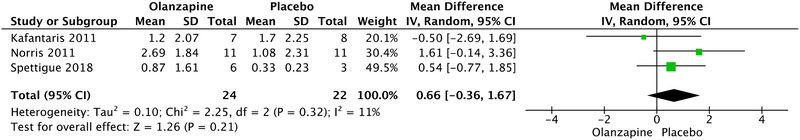
Effect of olanzapine as adjuvant treatment in adolescent

The effect of olanzapine as adjuvant treatment was examined in two studies. The increase in BMI was 0.66 kg/m^2^ (95%CI −0.36–1.67 kg/m^2^; *p *= 0.21; *I* ^2^ = 11%, *p* for heterogeneity = 0.32) (Figure 4). There was no obvious publication bias in all subgroup analyses on funnel plots (Supporting Information Figures S3–S5).

## 4 DISCUSSION

This article identified five double‐blind, randomized studies, including 304 patients with AN, showing that there were obvious differences in BMI at the end of treatment between groups, and the olanzapine group had a significant increase in BMI compared with the placebo group. This finding provides strong support for the efficacy of pharmacotherapy in adult AN. In a previous meta‐analysis, olanzapine showed a trend in the treatment of AN, but due to the limited sample size, the conclusion was blurred.(Dold et al, 2015; Rosager et al., 2021) In adolescents, although olanzapine as adjuvant treatment showed a trend to improving BMI, due to the limited number of participants enrolled, there was no significant difference. There was no heterogeneity in all subgroup analyses, which made the conclusion reliable.

An absolute cut‐off in terms of low BMI is not stipulated, since several other factors warrant consideration, including the patient's age, sex, BMI before the occurrence of symptoms, and rapidity of weight loss; however, a low weight (e.g., BMI ≤17.5 kg/m^2^) is usually observed in adults with AN. Olanzapine treatment resulted in an increase in BMI, 0.6 kg/m^2^, at the end of treatment. It was difficult to evaluate the absolute value of the increased BMI. The body weight changed more frequently in the olanzapine group than in the placebo group according to a study of 152 AN cases, and the mechanism of waves in BMI values during the treatment was still unclear (Attia et al., 2019). Olanzapine alone might be insufficient in the treatment of AN. An improved BMI is important in AN patients; cognitive remediation therapy (CRT), family therapy, and other nonpharmacological interventions also play important roles in the treatment of AN (Attia & Walsh, 2009; Fisher et al, 2018; Gan et al, 2021; Tchanturia et al, 2017).

Several limitations to this study must be considered when interpreting the results. Given the nature of AN as a rare disease, studies on AN can only enroll a limited number of patients, creating a substantial risk of selection bias. Second, the effects of olanzapine in subtypes of AN were not identified, because there were limited data on the effect of olanzapine in subcategories. Attia et al, 2019 reported there was no statistically significant differences by subtype. Third, the period of follow up was different in the studies examined. The efficacy of olanzapine was confirmed in 2–3 months, and its long‐term efficacy remains unclear.

## 5 CONCLUSION

Olanzapine showed efficacy in the treatment of AN, with weight gain at the end of treatment in adults. Its effect as adjuvant treatment in adolescents remains unclear. More comparative studies are needed.

## CONFLICT OF INTEREST

The authors declare no conflict of interest.

## AUTHOR CONTRIBUTIONS

RH and HC were involved in data acquisition and drafting the manuscript. HC contributed to study conception. All authors performed data acquisition, analysis, interpretation, and drafting. HC involved in interpretation and critical revision. All authors provided final approval and take accountability.

### PEER REVIEW

The peer review history for this article is available at https://publons.com/publon/10.1002/brb3.2498

## Supporting information

Supporting information

## Data Availability

The raw data are available by email on reasonable request to the corresponding author. E‐mail: chinsmd@gmail.com
