# Supplementary material for: Effectiveness of olanzapine in the treatment of anorexia nervosa: A systematic review and meta‐analysis
Source: Brain Behav. 2022 Jan 12;12(2):e2498. doi: 10.1002/brb3.2498 (PMC8865148; doi:10.1002/brb3.2498)
Supplement: Supplementary file 1 — Supporting information [file BRB3-12-e2498-s001.docx]

**Title:** Effectiveness of olanzapine in the treatment of anorexia nervosa: a systematic review and meta-analysis

Running title: Olanzapine in anorexia nervosa

Ruijun Han^1^, Qingtao Bian^1^, Hao Chen^2^

^1^ Department of Psychiatry, Beijing Huilongguan Hospital, Beijing 100096，China

^2^ Department of Internal Medicine, Teikyo University Hospital, Tokyo 173-8606, Japan

Corresponding author: Hao Chen

Department of Internal Medicine, Teikyo University Hospital, 2-11-1 Kaga, Itahashi, Tokyo 173-8606, Japan

E-mail: chinsmd@gmail.com

Tel: 03-3964-1211

Fax: 03-3964-1211

**Supplementary Figures**

Supplementary Fig. 1 Effect of olanzapine in increased BMI

Supplementary Fig. 2 Effect of placebo in increased BMI

Supplementary Fig. 3 Funnel plots for the difference in BMI of olanzapine versus placebo at the end of treatment

Supplementary Fig. 4 Funnel plots of increased BMI after treatment with olanzapine and placebo

Supplementary Fig. 5 Funnel plots of the effect of olanzapine as adjuvant treatment in adolescents
